# Cellulose as an adhesion agent for the synthesis of lignin aerogel with strong mechanical performance, Sound-absorption and thermal Insulation

**DOI:** 10.1038/srep32383

**Published:** 2016-08-26

**Authors:** Chao Wang, Ye Xiong, Bitao Fan, Qiufang Yao, Hanwei Wang, Chunde Jin, Qingfeng Sun

**Affiliations:** 1 School of Engineering, Zhejiang A & F University, Lin’an 311300, PR China; 2 Key Laboratory of Wood Science and Technology, Zhejiang Province, PR China

## Abstract

The lignin aerogels that are both high porosity and compressibility would have promising implications for bioengineering field to sound-adsorption and damping materials; however, creating this aerogel had a challenge to adhesive lignin. Here we reported cellulose as green adhesion agent to synthesize the aerogels with strong mechanical performance. Our approach—straightforwardly dissolved in ionic liquids and simply regenerated in the deionized water—causes assembly of micro-and nanoscale and even molecule level of cellulose and lignin. The resulting lignin aerogels exhibit Young’s modulus up to 25.1 MPa, high-efficiency sound-adsorption and excellent thermal insulativity. The successful synthesis of this aerogels developed a path for lignin to an advanced utilization.

In recent years, there has been increasing interest in the utilization of bio-renewable feedstocks as advanced materials. Aerogels are promising candidates for various advanced applications^1^,^2^,^3^,^4^,^5^ as a result of their low densities, small pore sizes and high-surface areas of the internal structures. However, the utilization of typical cellulose aerogels, a good natural renewable material^6^,^7^, was hampered by their poor mechanical performance. Therefore, there was the issue that found renewable raw materials to the preparation of aerogel with a strong mechanical performance.

Lignin, the second most abundant biopolymer after cellulose^8^, was an interesting candidate. It has a rigid, hyperbranched macromolecular structure composed of three different types of phenylpropane units and various functional groups, such as ether, hydroxyl, methoxy, aldehyde, and ester groups^9^,^10^,^11^,^12^,^13^. It is massively produced as a byproduct from papermaking and emerging cellulosic ethanol industries, and more than 98% of this material is directly poured into nearby waters or burned in an energy-recovery step^14^. So far, most of the applications of lignin still remain at a relatively low and rural level due to it not miscible with most polymers and even exhibiting deteriorated mechanical properties after blending^15^. In plant cell walls, the use of high efficiency and precision of lignin owed to regular and proper connection with other components, especially cellulose^16^,^17^,^18^.

Cellulose, the most abundant renewable natural polymer^19^,^20^, possesses many useful features, such as hydrophilicity, biocompatibility, hydroxyl reactivity, and reasonable thermal and mechanical stabilities^21^,^22^. Meanwhile, it also can be used as a green and renewable adhesion agent due to much hydroxyl group intro-molecule^23^. In the applications, dissolving cellulose is a key fabrication step. The room temperature ionic liquids (RTILs), a new class of solvent with high solubility of cellulose and lignin, can simply and straightforwardly gain the nanoscale of them without the harsh conditions and high energy consumption^24^,^25^,^26^. Compared with dissolution of cellulose, there was the fewer report on dissolution of lignin in RTILs, and there is almost no report on simultaneous dissolution of lignin and cellulose.

Herein, in the current paper, we employed 1-butyl-3-methylimidazolium chloride (BMIMCl), a common RTILs, as the solvent of simultaneous dissolution for the synthesis lignin aerogel with the cellulose as an adhesion agent. The wet gels were produced by simple solution-gelation chemical approach. After solvent exchange processing and further freeze drying, the obtained aerogels possessed strong mechanical performance, high-efficiency sound-adsorption, excellent thermal insulativity and high special surface area.

## Results

### Formation of lignin aerogels of cellulose as adhesion agent

**Figure 1:**
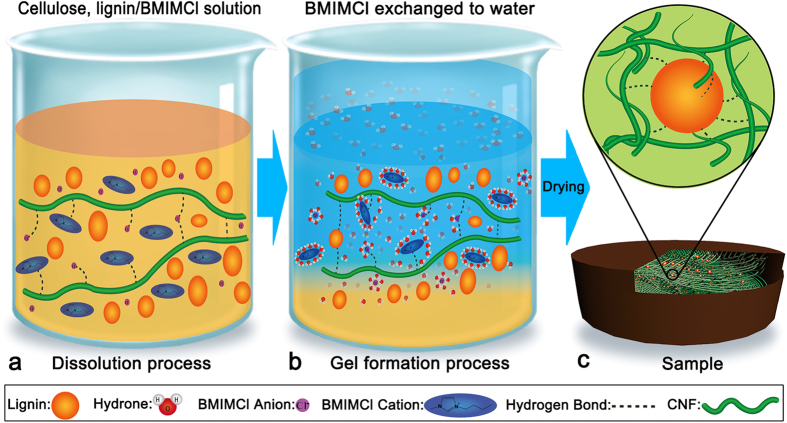
Schematic illustration of nanostructural organization formation process.

As shown in Fig. 1a, cellulose nanofibers and lignin particles dispersed in the ionic liquids which are composed of the anions and cations. They are respectively represented by a spherical particle and an ellipsoid with a polar headgroup (the imidazolium ring)^27^,^28^. Figure 1a represents the dissolved state of the starting cellulose, lignin/BMIMCl solution. The anions and cations can form hydrogen bonds with linear cellulose molecules to destroy the hydrogen bonds between the cellulose nanofibers^24^,^28^. In this process, anions and cations of the BMIMCl also form electro donor-acceptor (EDA) with the oxygen and hydrogen atoms of the C-6 and C-3 hydroxyl groups of adjacent cellulose chains^29^. These interactions result in the separation of different nanofibrils of cellulose and make it dissolved in the ionic liquids.

The gel formation process was shown in Fig. 1b. The formation of wet gels lies in the interactions between ions and the molecules of water. This process, either a cation or an anion attracted water molecules to its immediate vicinity. The negative (oxygen) side of a dipolar water molecule attracts and is attracted by cation in solution. Because of this ion-dipole force, water molecules cluster around cations, as shown in Fig. 1b. Similarly, the positive (hydrogen) ends of water molecules are attracted to anions. Thus, the hydrogen bonds between hydroxide radical from cellulose molecule chain and chloride ion from BMIMCl were destroyed by hydration of ions, causing bareness of the hydroxide radical in solution. The water molecule as well interacted with cations or anions acting on lignin molecule, and then large number of hydrogen bonds between cellulose and lignin produced. Meanwhile, some regeneration of cellulose, that is formation of cellulose intermolecular hydroxide radical, also made lignin particles embraced by cellulose nanofibers (CNF). The subsequent freeze drying step can avoid the collapse of pores and preserve largely the solid network formed in wet gels. Figure 1c shows the schematic illustration of nanostructure of samples after drying, in which lignin particles enclosed by cellulose nanofibers and formed hydrogen bond with them.

**Figure 2:**
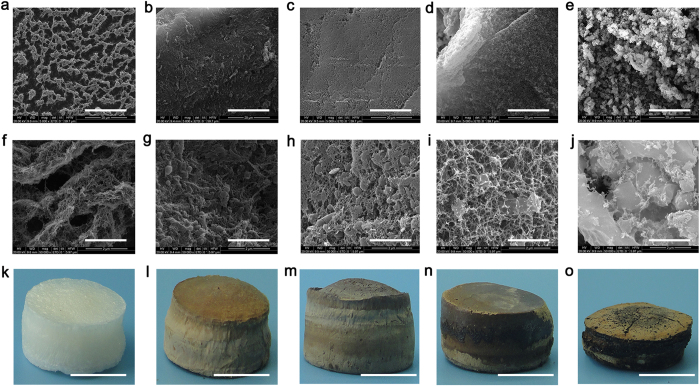
SEM images and photographs of samples. S-0: pure cellulose (**a,f,k**), S-50: 50% lignin (**b,g,l**), S-75: 75% lignin (**c,h,m**), S-90: 90% lignin (**d,i,n**), S-95: 95% lignin (**e,j,o**). Magnification: ×5,000 (**a–e**) and ×50,000 (**f–j**). Scale bars, 20 μm (**a–e**), 2 μm (**f–j**), 1 cm (**k–o**).

The pure cellulose aerogels (S-0) was compared with the other samples. Macroscopically, in addition to S-95 (Fig. 2o), the other samples possess good integrity and well-defined shapes without any cracks. However, the degree of color of these aerogels is suited well with the content of lignin. By changing the proportion of lignin from 0% to 95%, degree of the color gradually deepened (Fig. 2k–n), and it is not worth noting that there has been a distinct shriveling cellulose aerogels with 95% of lignin. On the macroscopic shape depend on the microscopic morphology.

The distinct morphologies were shown by scanning electron microscopy (SEM) images for samples with different content of lignin. Figure 2a,f, show irregularly shaped microscale and nanoscale pores of S-0, typically regenerated cellulose aerogels, at the different magnification. Under high magnification (×50,000), it is shown that aggregated and interconnected CNF make up the thick walls of the macroscopic. A transition to denser morphologies appears by adding the ratio of lignin in cross-linked network of cellulose. Figure 2, low magnification (×5,000), shows the pores become smaller, and even cannot be found in S-50 and S-75, respectively. Under high magnification (×50,000), the images clearly show nanoscale pores enclosed by CNF, and lignin granules were adhered by them (Fig. 2g,h). The lignin, mainly filling in the larger pores, contributes to decreasing of the porosity, pore size, and special surface area as compared to that in S-0 (Table 1). With the content of lignin increase to 90%, the size of pores and distribution of lignin particles are relatively uniform. S-95, filled in 95% of lignin, contains mass lignin particles observed by SEM (Fig. 2e). Consistent with the shrink of macro shape and the suddenly increase of density (Table 1), the three-dimensional (3D) network composed of CNF was broken (Fig. 2j).

### Characterization of physical properties

**Figure 3:**
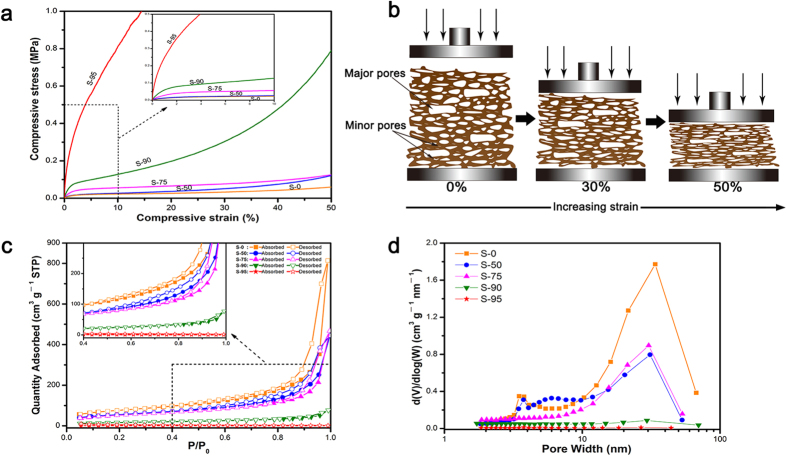
(**a**) Compressive stress-strain curves for the samples (5 mm thick). (**b**) Schematic description of the changes in cellular structure with compressive deformation. **(c)** Nitrogen adsorption and desorption isotherms. (**d**) BJH desorption pore distribution of the samples.

Figure 3a shows the compressive stress-strain curves of all samples with thickness of 5 mm up to 50% strain. In addition to S-95, other samples display a small linear elastic region about 2%. The sample mixed 95% lignin, showing dramatic increase with strain, has a Young’s modulus up to 25.1 MPa, which is 20 times above S-0 as compare. But beyond the yield point, all curves show a plateau where the stress rises tardily with growing compressive strain, indicating that the porous structure in samples collapsed increasingly under the compressive load. Toward higher strains, all samples show a soaring increase in stress due to the densification of samples after complete collapse of the porous structure. Figure 3b presents the strain is absorbed at a high level by the deformation of major cellular pores and minor pores^30^,^31^. All samples did not breakage until the force transducer of the testing machine reached its setting limit, although the deformation of specimens was irreversible. The advantage is lack in common inorganic counterparts^32^. The stress-strain curves suggest that mixing the lignin in cellulose can improve compression resistance.

The specific surface area of all samples (S_BET_), calculated from the Brunauer-Emmett-Teller (BET) equation, decreases from 268.9 m^2^ g^−1^ for S-0 to 5.1 m^2^ g^−1^ for S-95 (Table 1). The nitrogen adsorption-desorption isotherms of the S-0, S-50 and S-75 have a distinct hysteresis loops observed in the relative pressure range of 0.4‒1.0, and are of type IV according to IUPAC classification (Fig. 3c). A small hysteresis loop, as well corresponding to the presence of mesoporous structures, of S-90 can be observed in Fig. 3c. Meanwhile, there is no a loop in the nitrogen adsorption-desorption isotherms of S-95, which agreed well with its morphologies and microstructures. The pore width distribution curves from the desorption branch by the Barrett-Joyner-Halenda (BJH) method of samples were plotted Fig. 3d. It is shows that the samples contain pores ranging from 1 nm to 100 nm, and these pores have pore diameter in the range of 8.0‒17.4 nm and total pore volumes to 0.015‒1.251 cm^3^ g^−1^ (Table 1). The distinct peaks, in the range of 10‒30 nm, can be observed in pore diameter distribution of S-0, S-50, and S-75. Furthermore, the two former can be found that possess well sub-peak about 3.5 nm. Peak of the other one is relatively concentrated, which explains that its pore width is larger than S-50. About 30 nm, the peak of S-90 is almost negligible. Obviously, S-95 is the lack of mesoporous structure, also indicated by their SEM images at high magnification.

### Characterization of functional group

**Figure 4:**
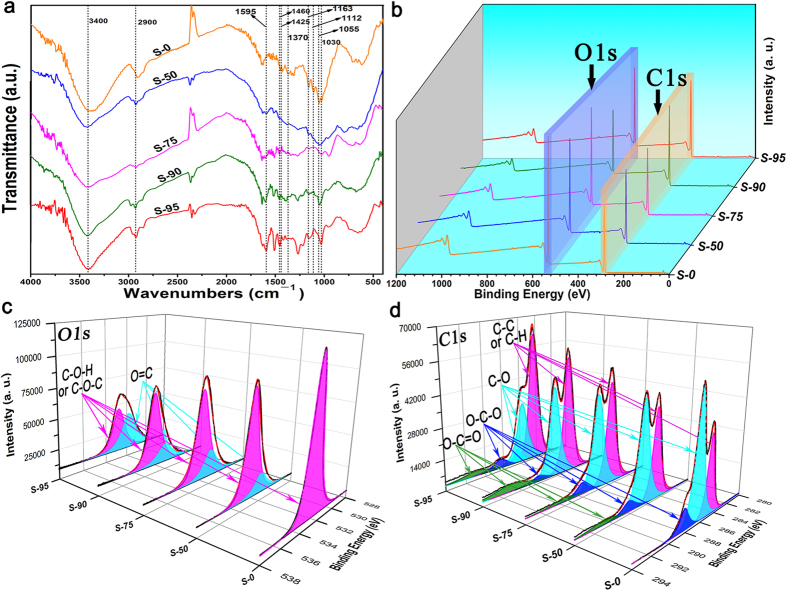
(**a**) Comparison of FTIR spectra of samples. (**b**), **(c**), and (**d**) are the XPS spectra of samples for an overview spectrum, O 1s spectrum, and C 1s spectrum.

As shown in Fig. 4a, five FTIR spectra come from the samples mixed different content of lignin. All samples exhibited the characteristic bonds of cellulose at 2900, 1370, 1163, and 1055 cm^−1^ separately, which corresponded to the C-H stretching and deformation modes, the C-O-C symmetric and antisymmetric stretching and the C-O stretching modes^33^,^34^,^35^. After this mixed lignin, the unchanged spectral signature of cellulose reveals a good stability of the samples. Besides of the FTIR spectra of S-0, the others can be observed the appearance of different characteristic peaks: one relative to the aromatic skeletal vibration and C=O stretching located at 1595 cm^−1^, evidently, resulting from the lignin^36^,^37^.

To investigate the chemical composition of samples and determine the chemical status of O and C elements, they were measured by X-ray photoelectron spectroscopy (XPS). Figure 4b presented the survey scan of XPS spectra, and showed that all samples contain O and C elements and theirs corresponding photoelectron peaks were O 1s and C 1s, respectively. Figure 4c depicted the O 1s regions XPS spectrum of all samples. In S-0, pure cellulose, the well peak of O 1s at 532.9 eV could attributed to C-O*-H or C-O*-C^38^ (Table 2). In other samples containing lignin, the O 1s core levels were shifted to lower energy and fitted into two peaks, demonstrating that different kinds of O binding states were existed. The higher peaks remained stable, and the lower peaks located at about 531.9 eV and was assigned to O*=C^39^. The high resolution XPS spectrums of the C 1s were exhibited in Fig. 4d. The regions of C 1s were fitted into distinct components corresponding O-C*=O, O-C*-O, C*-O, C*-C* and C*-H bonds in samples. But the O-C*=O bond only was found in the samples mixed lignin. Furthermore, the intensity of C*-C* or C*-H bonds increase gradually with the content of lignin, consistent with atomic ratio C/O (Table 3).

### Characterization of sound-absorption and thermal insulation

**Figure 5:**
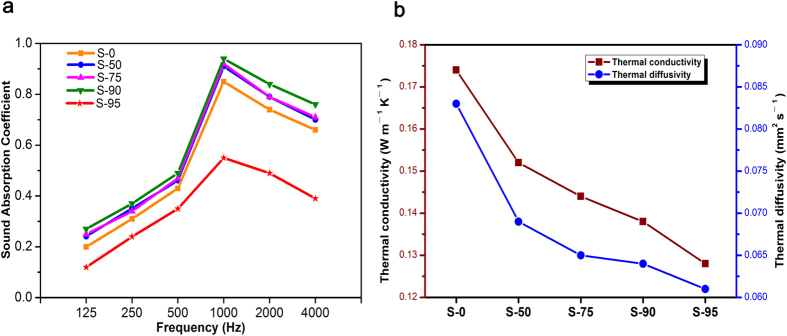
(**a**) The sound-absorbing performance testing of samples. (**b**) The thermal conductivity and diffusivity of samples.

The aerogels and woody materials possessed the interesting performance of sound absorption or dissipation^40^,^41^. Figure 5a showed the variation of sound absorption coefficient of lignin aerogels as a function of frequency. The sound absorption effect of all samples is better in the medium-high frequency, and had a distinct increasing at the frequency range 125‒1000 Hz and slight decreasing range 1000‒4000 Hz. In addition to S-95, sound absorption coefficients of other samples contain lignin have a small rise with the maximum of 0.94, compared with S-0. The results were attributed to the high porosity and small pore size^42^. Acoustic waves moved through lignin porous aerogels and then were efficiently dissipated because they entered through these pore channels and were converted into heat by viscous and thermal losses^43^. The weakening of sound absorption characteristic of S-95 result from its porosity plummeting.

As illustrated in Fig. 5b, the S-0 exhibited a well thermal conductivity of 0.174 W m^−1^ K^−1^ by a heat flow meter at atmospheric pressure. The result was attributed to high porosity of the cellulose aerogels^44^ (98.4%). Despite the porosity of samples decreasing, it was worth note that promoting of thermal insulation performance of lignin aerogels corresponded with increasing of content of lignin, and the similar change was presented in thermal diffusivity curves (Fig. 5b). In the S-95, the thermal conductivity and diffusivity respectively decreased to 0.128 W m^−1^ K^−1^ and 0.061 mm^2^ s^−1^. Benefiting from the lignin intrinsical the thermal stability^45^,^46^, heat insulation property^47^ and a relative high porosity, the thermal insulation performance of samples was estimated to be one order of magnitude greater than that of commonly used insulation materials^48^ (Table 4), and these results supported the thermal insulation of the materials.

## Discussion

Lignin aerogels of cellulose as adhesion agent were successfully prepared by dissolving cellulose and lignin powders in BMIMCl, regenerating in the deionized water and freezing drying process. It was observed that CNF can form hydrogen bond with lignin particles for synthesis of aerogels. The strengthening of mechanical performance and increasing of density were correlated with the content of lignin^49^,^50^, which is considered to use as an advanced material to prepare aerogels.

In conclusion, we have demonstrated the detailed formation mechanism for the simple synthesis of lignin aerogels of cellulose as adhesion agent through the solution-gelation approach. The unmodified lignin, applications of relatively low and rural level^51^, was reconstructed into 3D lamellar porous aerogels with tunable special surface area and densities via adhesion of CNF. With their low density, strong mechanical performance and functionality in terms of sound absorption and thermal insulation, we envision that the samples will open up numerous opportunities for a range of applications in dampers, heat insulators, sieves, absorbents and tissue engineering scaffolds.

## Methods

### Materials

Cellulose and lignin (Sigma-Aldrich Co. ltd, USA) were analytical grade and used as received. The BMIMCl was purchased from Shanghai Boylechem Co. ltd (Shanghai, China). Other chemicals were used as received without further purification.

### Preparation of lignin aerogels of cellulose as adhesion agent

**Figure 6:**
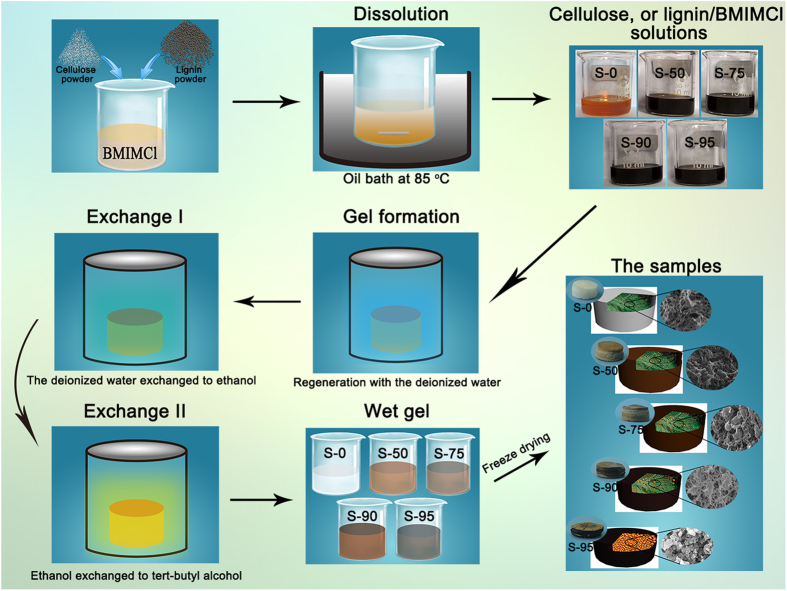
Schematic presentation of samples preparation.

The detailed fabrication process is described in Fig. 6. Cellulose powders (0.2 g) and different weight of lignin powders (0.0 g, 0.2 g, 0.6 g, 1.8 g, 3.8 g) were mixed with 9.8 g of BMIMCl in a 25 mL beaker. The beakers were immersed in an oil bath at 85 °C via magnetic stirring for 3 h to form a homogeneous solution. The homogeneous viscous solutions were transferred to 10 mL beakers to obtain a defined thickness. Then, all samples were immersed in the first regeneration bath with the deionized water. The bath was replenished at least thrice until Cl^−^ was not detected using AgNO_3_ solution. Next the gels respectively were immersed with absolute ethanol and tert-butyl alcohol to obtain more homogeneous cellular structure. The immersion time in each bath, which was replenished at least thrice, was longer than 6 h. After undergoing several exchange processes, the wet gels (tert-butyl alcohol) were freezed drying by refrigerator at −50 °C for more than 48 h.

### Characterization

The morphology was observed for samples sputter-coated with gold by using a Quanta^TM^–250 field-emission SEM (FEI, USA) at an accelerating voltage of 20 kV. Nitrogen adsorption-desorption isotherms were measured at 77 K with a Micromeritics ASAP 2020 surface area and porosity analyzer (Micromeritics instrument Ltd., USA). Sound-absorption tests were carried out a JIZB standing wave tube absorption coefficient test system (Beijing JT Technology Co. ltd., China). The thermal conductivity of samples with 5 mm thickness was obtained by using Netzsch LFA 427 under ambient conditions.

Compression tests were carried out on a CMT–6104 electromechanical universal testing machine (MTS systems (China) Co. ltd). During the test, the samples with thickness of 5 mm were compressed with a speed of 1 mm min^−1^ until reaching a maximum load of 1 kΝ. Three specimens were measured for each sample.

The density of the sample (ρ_S_) was calculated from its weight (g) and volume (cm^3^). The porosity of the sample (P) was calculated by the following equation (1), where (ρ_C_) and (ρ_L_) respectively is the density of bulk cellulose (1.528 g/cm^3^) and lignin (1.300 g/cm^3^), *n* is the quality percentage of cellulose for lignin.


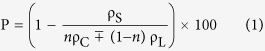


## Additional Information

**How to cite this article**: Wang, C. *et al*. Cellulose as an adhesion agent for the synthesis of lignin aerogel with strong mechanical performance, Sound-absorption and thermal Insulation. *Sci. Rep.* **6**, 32383; doi: 10.1038/srep32383 (2016).

## Figures and Tables

**Table 1:** Physical properties of all samples.

Sample^A^	density (g cm^−3^)	porosity (%)	S_BET_ (m^2^ g^−1^)	pore volume^B^ (cm^3^ g^−1^)	pore size^B^ (nm)	Young’s modulus (MPa)	Compressive stress at 50% strain^C^ (MPa)
S-0	0.024	98.4	268.9	1.251	17.4	1.1	0.06
S-50	0.048	96.6	198.3	0.682	12.7	1.2	0.12
S-75	0.052	96.2	190.6	0.709	15.7	2.8	0.13
S-90	0.080	94.0	58.2	0.116	8.3	5.9	0.79
S-95	0.403	69.3	5.1	0.015	8.0	25.1	5.46

^A^Number in the code refers to the lignin content used in the sample (wt%): S-0: pure cellulose, S-50: 50% lignin, S-75: 75% lignin, S-90: 90% lignin, S-95: 95% lignin.

^B^Obtained from desorption isotherms by BJH method.

^C^For samples with 2.5 mm thick.

**Table 2:** Assignments of Binding Energies (eV) of Main XPS Regions.

	S-0	S-50	S-75	S-90	S-95	assignment
O 1s 1	532.9	533.0	532.8	533.1	532.8	C-O*-H and C-O*-C
O 1s 2	—	531.9	531.9	532.0	531.8	O* = C
C 1s 1	—	290.5	290.6	291.2	290.9	O-C* = O
C 1s 2	288.1	288.2	288.1	288.1	288.3	O-C*-O
C 1s 3	286.5	286.5	286.1	286.3	286.3**/**285.7	C*-O
C 1s 4	284.8	284.8	284.6	284.6	284.3	C*-C* or C*-H

**Table 3:** Atomic ratio C/O computed from XPS data.

Al Kα	S-O	S-50	S-75	S-90	S-95
C/O	1.8	2.2	2.4	2.8	3.1

**Table 4:** Thermal insulation properties of samples and other commonly used thermal insulation materials.

Materials	Density (g cm^−3^)	Thermal conductivity (W m^−1^ K^−1^)	Diffusivity (mm^2^ s^−1^)	Source
S-75	0.052	0.144	0.065	—
S-90	0.080	0.138	0.064	—
S-95	0.403	0.128	0.061	—
Lightweight concrete	0.551	0.155	0.319	48
Polyurethane board	0.028	0.024	0.558	48
	0.033	0.022	0.434	
Class fiber (axial)	0.030	0.042	1.460	48
	0.095	0.038	0.417	
Rock wool (axial)	0.050	0.042	1.000	48
	0.120	0.040	0.397	
